# Left Internal Thoracic Artery versus Saphenous Vein Grafts to Left Anterior Descending Artery after Isolated Coronary Artery Bypass Surgery †

**DOI:** 10.3390/life14030385

**Published:** 2024-03-14

**Authors:** Suvitesh Luthra, Hannah Masraf, Mostafa Elbadry Mohamed, Pietro G. Malvindi, Davorin Sef, Szabolcs Miskolczi, Theodore Velissaris

**Affiliations:** 1 Wessex Cardiothoracic Centre, University Hospital Southampton, Southampton SO16 6YD, UK; s.luthra@soton.ac.uk (S.L.); mostafa.mohamed@uhs.nhs.uk (M.E.M.); szabolcs.miskolczi@uhs.nhs.uk (S.M.); theodore.velissaris@uhs.nhs.uk (T.V.); 2 Human Development and Health, Faculty of Medicine, University of Southampton, Southampton SO17 1BJ, UK; 3 Division of Surgery, Kingston Hospital NHS Foundation Trust, Kingston upon Thames KT2 7QB, UK; hannah.masraf1@nhs.net; 4 Department of Cardiothoracic Surgery, Faculty of Medicine, Assiut University, Assiut 71111, Egypt; 5 Cardiac Surgery Unit, Lancisi Cardiovascular Center, Ospedali Riuniti delle Marche, Polytechnic University of Marche, 60126 Ancona, Italy; pg.malvindi@hotmail.com; 6 Department of Cardiac Surgery, University Hospitals of Leicester, Leicester LE5 4PW, UK

**Keywords:** left internal thoracic artery, saphenous vein graft, coronary artery bypass surgery, arterial grafts

## Abstract

Background: This study compared perioperative outcomes and long-term survival of saphenous vein grafts (SVGs) versus left internal thoracic artery (LITA) to left anterior descending artery (LAD) in isolated coronary artery bypass graft surgery (CABG). Methods: In this retrospective, single-centre study, we included patients with primary isolated CABG from January 2001 to July 2022. Baseline demographics were compared between SVG-LAD and LITA-LAD. Univariable and multivariable regressions were performed for predictors of in-hospital death. Propensity score matching was performed for LITA-LAD vs. SVG-LAD. Kaplan–Meier survival curves were generated for comparison of survival. Cox proportional hazards model was used for predictors of survival. Results: A total of 8237 patients (1602 SVG-LAD/6725 LITA-LAD) were included. Median age was 67.9 years (LITA-LAD; 67.1 years vs. SVG-LAD; 71.7 years, *p* < 0.01). A total of 1270 pairs of SVG-LAD were propensity-matched to LITA-LAD. In matched cohorts, in-hospital mortality (0.8% vs. 1.6%, LITA-LAD and SVG-LAD respectively; *p* = 0.07), deep sternal wound infection, new cerebrovascular events, renal replacement therapy and hospital stay >30 days were similar. SVG-LAD did not adversely affect in-hospital mortality (OR; 2.03, CI; 0.91, 4.54, *p* = 0.08). Median long-term survival was similar between the groups (13.7 years vs. 13.1 years for LITA-LAD and SVG-LAD respectively, log rank *p* < 0.31). SVG-LAD was not a predictor of adverse long-term survival. (HR; 1.06, 95% CI; 0.92, 1.22, *p* < 0.40). Long-term survival was better with LITA-LAD for LVEF <30% (log rank *p* < 0.03). Conclusions: There was no difference in the propensity-matched cohorts for use of SVG vs. LITA to the LAD. Further contemporary long-term studies are needed for substantiation.

## 1. Introduction

The concept of survival benefit associated with left internal thoracic artery (LITA) grafting to the left anterior descending (LAD) artery has been fundamental to surgical revascularisation for over four decades now [1,2,3,4,5]. It forms the basis for revascularisation guidelines for coronary artery disease, benchmark quality indicators for surgical revascularisation and the grounds for recommendations for surgical revascularisation as opposed to percutaneous coronary interventions (PCI) in the left main stem, left main stem equivalent, proximal LAD and severe three-vessel coronary artery disease [6]. The concept has remained unchallenged and enshrined in surgical practice, despite the advances in drug-eluting stents, greater secondary cardiovascular prevention strategies with the use of statins, dual antiplatelet agents and control of risk factors like diabetes mellitus and improved vein graft patency [6].

The LITA-to-LAD graft is the only one associated with the survival benefit of surgical revascularisation [5]. We hypothesized that survival after surgical revascularisation is independent of the type of conduit to the LAD, if the LAD territory has been reasonably and adequately revascularized. 

The aim of this study was to compare outcomes of saphenous vein grafts (SVG) to LITA grafts for the LAD in isolated coronary artery bypass graft surgery (CABG). The objectives of the study were to

1. Compare perioperative outcomes and long-term survival in patients with SVG versus a LITA to the LAD.
2. To compare and identify predictors of long-term survival, especially the influence of grafting strategy for the LAD.

## 2. Materials and Methods

### 2.1. Study Design and Data Collection

This is a retrospective, single-centre, observational study conducted as per the STROBE guidelines (Supplementary Table S1—STROBE checklist). Institutional board approvals were obtained for use of patient data in compliance with local data protection policies (Safeguard no—7498, 10 March 2023). Consent was waived due to the nature of the study and prior consent at the time of surgery. Patients who had opted out from data sharing were not included in the study.

Data were collected for all patients who underwent first-time isolated CABG (January 2001 to July 2022). Perioperative data were collected from the institutional database (Patient Administration System, e-CAMIS, Yeadon, Leeds, UK) validated by the National Institute of Health Outcomes Research (NICOR). Long-term survival data were obtained from a combination of the Patient Administration System (e-CAMIS) and the NHS Spine Portal Summary Care Records (SCR), which is the GP electronic database. Survival data were 100% complete.

### 2.2. Inclusion and Exclusion Criteria

We included all patients with first-time isolated CABG who had either LITA or SVG to the LAD. All patients with other concomitant procedures, complex composite grafting, emergency or salvage surgery and previous sternotomy or redo-CABG were excluded.

### 2.3. Surgical Technique

LITA was harvested as a pedicle and SVGs were harvested with open or endoscopic techniques. All surgeries were performed using a standard median sternotomy. CABG was performed in a standard fashion as per surgeon’s preference for technique. LITA was our preferred conduit for grafting the LAD. SVG was used instead of LITA for grafting the LAD due to various technical or anatomical reasons, such as insufficient size or length, injury or dissection during harvesting, poor flow, chest wall adhesions, concerns for chest wall bleeding and in elderly patients. 

### 2.4. Statistical Analysis

Baseline demographics included 15 variables (Table 1) previously described for EuroSCORE (European System for Cardiac Outcomes Risk Evaluation) among the data points used for the NICOR Adult Cardiac Surgery Database (ACSD) in the UK. Patients were divided into 2 groups—SVG-LAD and LITA-LAD. Categorical variables were compared using the chi-squared test and continuous variables were compared using the Mann–Whitney test. Univariable and multivariable regressions were performed for predictors of in-hospital death.

Propensity score matching was performed using logistic scores. Patients with LITA-LAD were posteriorly matched to SVG-LAD in a 1:1 ratio with the nearest neighbour method without replacement with a calliper width of 0.2 of the standard deviation of the logit of the propensity score. Balance was assessed for the standard mean differences (SMD). 

Univariable regression analysis was performed for predictors of in-hospital mortality. Variables with *p* < 0.20 were used in the multivariable analysis. Post-discharge survival was calculated from the date of discharge to death or last follow-up survival. The Cox proportional hazards model was used for calculation of hazard ratios. 

Pre-discharge categorical (dichotomized) and continuous variables, including the composite endpoint of re-exploration for bleeding/tamponade, new postoperative transient ischemic attack or stroke, hemofiltration, deep sternal wound infection, permanent pacemaker implantation and length of hospital stay (LOS) >30 days, were used for analysing predictors of long-term survival. Proportionality assumption was tested using Schoenfeld residuals. Kaplan–Meier survival curves were generated, and equality of survivor function was tested using a log rank (Mantel Cox) and Wilcoxon test. A subgroup survival analysis was performed for age (less than and over 70 years), logistic EuroSCORE (<10% or >10%), diabetes mellitus and left ventricular ejection fraction (LVEF) (<30% or >30%).

*p* value of <0.05 was considered statistically significant. The analysis was generated using Statistical Analysis Software (SAS), Version 3.8, SAS University Edition (SAS Institute Inc., Cary, NC, USA) and SPSS version 27 (Armonk, NY, USA).

## 3. Results

A total of 8237 patients (1602 SVG-LAD/6725 LITA-LAD) with isolated primary CABG were included. The baseline demographics of the unmatched and matched groups are given in Table 1. Median follow-up was 8.5 (IQR; 4.8, 13.2) years. 

Median age was 67.9 years (LITA-LAD; 67.1 years vs. SVG-LAD; 71.7 years, *p* < 0.01). The majority (73.2%) had three-vessel CAD. The SVG-LAD group had a significantly higher proportion of patients with chronic obstructive pulmonary disease (COPD), poor LVEF, neurological dysfunction, LMS disease, preoperative intra-aortic balloon pump and higher log EuroSCORE (5.08 vs. 3.71, *p* < 0.01).

**Figure 1:**
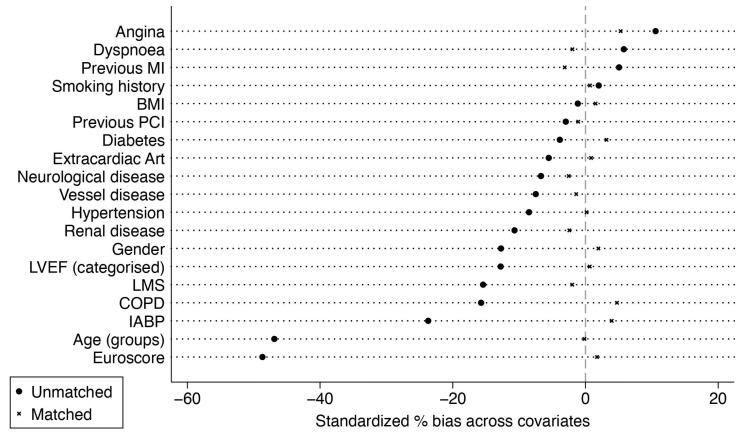
Standardized mean differences before and after matching. BMI, body mass index; COPD, chronic obstructive pulmonary disease; HTN, arterial hypertension, LVEF, left ventricular ejection fraction; LMS, left main stenosis; IABP, intra-aortic balloon pump; MI, myocardial infarction; PCI, percutaneous intervention.

In total, 1270 pairs of patients with SVG-LAD were propensity-matched to LITA-LAD. The covariate balance for standardized mean differences is shown in Figure 1.

### 3.1. Perioperative Results

In the matched cohorts, there was no difference in the cross-clamp time, but cardiopulmonary bypass time was significantly longer in the SVG-LAD group (Table 2). Rates of deep sternal wound infection, new cerebrovascular events, RRT and LOS >30 days were similar between groups (Table 3). A higher proportion of patients in the SVG-LAD group required re-exploration (1.1% vs. 2.3% for LITA-LAD and SVG-LAD, respectively, *p* = 0.02). In-hospital mortality was similar between the groups (0.8% vs. 1.6%, LITA-LAD and SVG-LAD, respectively; *p* = 0.07). 

Age, Canadian Cardiovascular Society (CCS) angina class 3–4, New York Heart Association (NYHA) dyspnoea class 3–4 and logistic EuroSCORE were predictors of adverse perioperative mortality (Supplementary Table S2). Use of SVG-LAD did not adversely affect in-hospital mortality (OR; 2.03, CI; 0.91, 4.54, *p* = 0.08). 

### 3.2. Survival

**Figure 2:**
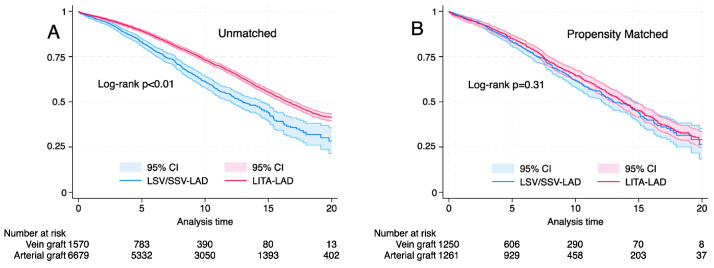
Kaplan–Meier graph showing the long-term survival based on conduit (left internal thoracic artery or saphenous vein graft). (**A**) Unmatched cohort, log rank *p* < 0.001, Wilcoxon; *p* < 0.001. (**B**) Propensity matched cohort log rank *p* = 0.31, Wilcoxon *p* = 0.35.

Median survival was 16.1 years (IQR; 15.6–16.5 years) and was significantly better for LITA-LAD in the unmatched cohort (16.5 years vs. 12.9 years for LITA-LAD and SVG-LAD, respectively, log rank *p* < 0.01, Wilcoxon *p* < 0.01). However, after propensity score matching, the long-term survival was similar between the groups (13.7 years vs. 13.1 years for LITA-LAD and SVG-LAD, respectively, log rank *p* < 0.31, Wilcoxon *p* < 0.34) (Figure 2).

Cox proportional hazards analysis showed preoperative renal failure (HR; 1.86, 95% CI; 1.33, 2.99, *p* < 0.01), LVEF < 30% (HR; 1.66, 95% CI; 1.32, 2.10, *p* < 0.01), smoking history (HR; 1.38, 95% CI; 1.18, 1.62, *p* < 0.01), arterial hypertension (HR; 1.22, 95% CI; 1.04, 1.42, *p* < 0.01), pulmonary hypertension (HR; 1.28, 95% CI; 1.07, 1.53, *p* < 0.01), extracardiac arteriopathy (HR; 1.22, 95% CI; 1.00, 1.49, *p* = 0.04), diabetes mellitus (HR; 1.68, 95% CI; 1.43, 1.98, *p* < 0.01), (HR; 3.66, 95% CI; 1.33, 2.99, *p* < 0.01) and age (HR; 1.09, 95% CI; 1.08, 1.10, *p* < 0.01) to be predictors of adverse long-term survival (Table 4). SVG-LAD was not a predictor of adverse long-term survival. (HR; 1.06, 95% CI; 0.92, 1.22, *p* < 0.40).

### 3.3. Subgroup Analysis

**Figure 3:**
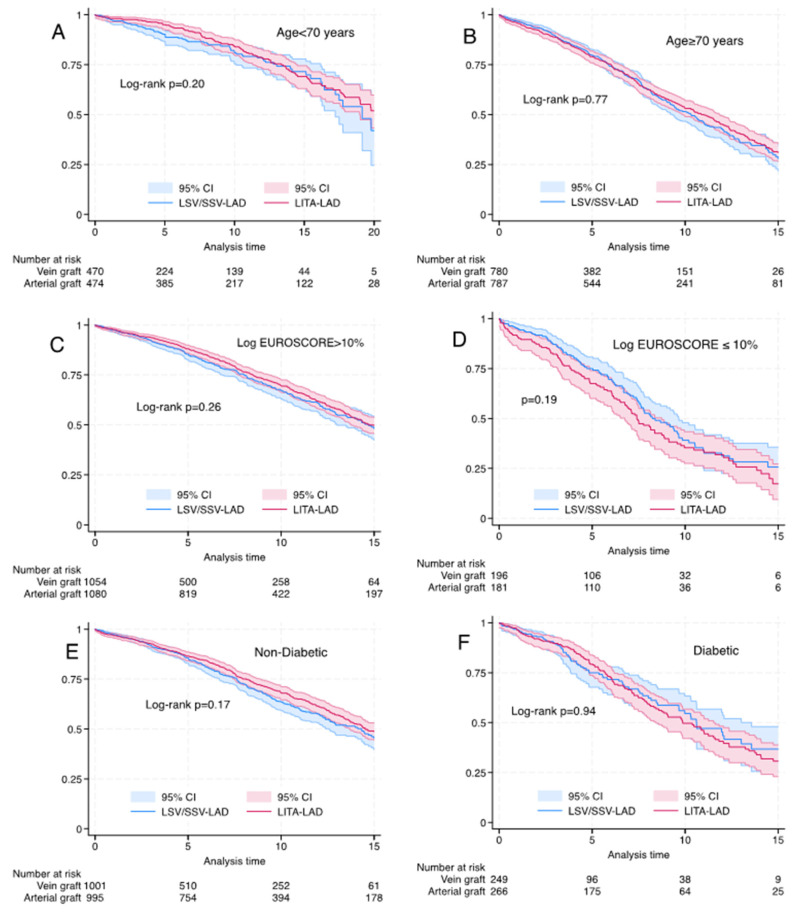
Kaplan–Meier graph for propensity-matched cohorts for left internal thoracic artery vs. saphenous vein graft to the left anterior descending artery). (**A**) Age <70 years, log rank *p* = 0.20, Wilcoxon *p* = 0.07. (**B**) Age > 70 years, log rank *p* = 0.77, Wilcoxon *p* = 0.897. (**C**) Log EuroSCORE < 10% log rank *p* = 0.26, Wilcoxon *p* = 0.16. (**D**) Logistic EuroSCORE > = 10%: log rank *p* = 0.19, Wilcoxon *p* = 0.11. (**E**) Non-diabetic patients, log rank *p* = 0.17, Wilcoxon *p* = 0.19. (**F**) Diabetic patients, log rank *p* = 0.59, Wilcoxon *p* = 0.94.

**Figure 4:**
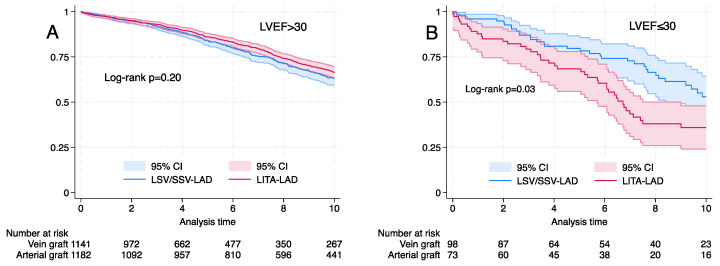
Kaplan–Meier graph for propensity-matched cohorts for left internal thoracic artery (LITA) vs. saphenous vein graft (SVG) to the left anterior descending artery (LAD). (**A**) Left ventricular ejection fraction (LVEF) >30%, log rank *p* = 0.20. (**B**) LVEF > 30%, log rank *p* = 0.03.

Among the matched cohorts, there was no difference in long-term survival with the use of SVG-LAD for subgroups of age (<70 and >70 years), logistic EuroSCORE (<10% and >10%) and diabetes mellitus (Figure 3). Long-term survival was better with LITA-LAD for LVEF <30% (log rank *p* < 0.03) (Figure 4).

## 4. Discussion

The belief in the survival benefit of a LITA-LAD graft is fundamental to surgical revascularisation and choice over PCI. LITA-LAD is the established gold standard and a benchmark quality indicator for CABG since the survival benefit demonstrated by Loop et al. almost four decades ago [7]. National Quality Forum has endorsed the use of LITA (NQF #0734 and operative care process domain 2 of the composite CABG score of NQF #0696) since 2012 as a quality metric for isolated CABG. There are financial disincentives for failure to use LITA-LAD during CABG, with no exceptions based on age or risk profile of the patients. Both the American Heart Association/American College of Cardiology and the European Association of Cardiothoracic Surgery guidelines recommend LITA to LAD as a Class 1B (Class of Indications—1 (strong), Level of Evidence—B-NR (nonrandomized studies and meta-analysis)) indication for isolated CABG [8,9]. SVGs are used in over 90% of surgical revascularisations and remain a versatile, reliable conduit. Evidence from the single randomized study (Arterial Revascularisation Trial) does not support the use of bilateral ITA, although previous meta-analyses of retrospective studies suggested better survival after up to 10 years of follow-up [10,11,12]. Uptake of bilateral ITA grafting has remained low despite recommendations for use in younger patients at low risk of sternal dehiscence.

LITA is usually precluded as a conduit of choice for the LAD due to size, length and poor flow; chest wall adhesions from a previous surgery; injury at harvest; concerns for chest wall bleeding; and absence of survival benefit in the elderly. For diffuse long segment disease and dilated hearts, the length might not be suitable for a distal LAD graft. Concerns regarding chest wall bleeding in the elderly, sternal dehiscence and to avoid opening the left pleural space in the presence of poor pulmonary functions may also preclude LITA. SVG can offer a faster operation, though it does need an additional proximal anastomosis to the aorta. In this analysis, higher risk elderly patients were most likely to have SVG-LAD.

There has been only one reported RCT comparing the use of SVG vs. LITA for the LAD [13,14]. This trial reported a significant mortality (SVG; 17.9% versus LITA; 7.7%, *p* < 0.05). Angiography revealed 76.3% patency of the SVG-LAD and 94.6% patency of the LIMA-LAD at 10 years. Cardiac-related mortality was 12.8% vs. 7.7% for SVG-LAD and LITA to LAD at 10 years. Hlatky et al. studied the adoption and effectiveness of LITA in 60,896 propensity-score-matched Medicare beneficiaries (median 6.8 year follow-up) [14]. LITA use was associated with lower all-cause mortality (adjusted hazard ratio 0.77, *p* < 0.001), lower death or myocardial infarction (adjusted hazard ratio 0.77, *p* < 0.001) and fewer repeat revascularisations over a 5-year follow-up (8% vs. 9%, *p* < 0.001). The association between LITA use and lower mortality was significantly weaker (*p* ≤ 0.008) for older patients, women and for patients with diabetes mellitus or peripheral arterial disease.

Our study raises very fundamental questions against contemporary beliefs of superiority of arterial grafts over vein grafts. Graft patency is not just a matter of conduits but also the territories grafted. LITA, unlike the SVG, has higher endogenous production of the vasodilatory nitrous oxide, which is believed to improve graft patency. LITA also has an adaptive morphological and rheological response after grafting to LAD that improves patency [15]. One of the explanations could be that the presumed greater patency of LITA is not due to the conduits per se, but is also, in part, dependent on the territory it is grafted to, i.e., LAD [16]. Graft patency for ITA is lower when grafted to circumflex/right coronary territories. Graft patency for SVG is also higher for LAD territory compared to other territories. The Veterans Affairs (VA) study reported that for SVGs to the LAD, the 10-year patency was 90% for vessels >2.0 mm versus 52% for vessels <2.0 mm (*p* < 0.001) [17]. For the LITA, the 10-year patency was 100% for vessels >2.0 mm versus 82% for vessels <2.0 mm (*p* = 0.008) [17]. In the contemporary COMPASS study, CT angiography revealed graft failure in 6.4% (68/1068) of cases for LIMA, 9.9% (9/91) for radial artery, 10.4% (232/2239) for saphenous veins at 1 year, and 26.8% (22/82) for RIMA grafts [18]. SVG patency is equivalent to radial arteries, although remains below LITA [18]. Also, it may be possible that SVG to LAD, with a relatively larger vascular bed and greater run-off, may have better patency compared to similar grafts to other territories with lower run-off. 

The survival benefit accrues from only the LITA-LAD graft. It is an axiomatic presupposition that graft patency equates to longer survival. This seems to be true for the LAD territory alone as opposed to territories of the circumflex and dominant right coronary arteries where revascularisation by surgical or nonsurgical means primarily serves only to ameliorate angina but offers no survival benefit. It could be argued that the findings of this study reinforce the results of the Arterial Revascularisation Trial (ART). ART showed in a randomized control setting that there is no difference between using bilateral ITA compared to a single ITA, meaning that for the second-best conduit, there is no survival difference between a SVG and an ITA to a non-LAD territory (since all patients had ITA graft to the LAD) [19]. Our study would now seem to suggest (albeit in a nonrandomized setting) that there may be no difference between an SVG and LITA, even in the LAD territory. The reasons, however, differ from those in ART. It is also likely that SVG patency has improved over time. The understanding of vein graft failure has improved substantially [20]. Better harvesting techniques, use of statins, optimal medical therapy for coronary disease and dual anti-platelets have possibly improved patency rates for SVG over the last two decades.

This limited study by no means establishes an equivalency between arterial and vein grafts to the LAD. However, it shows that in selected patients, in the absence of a usable LITA or relative contraindications to LITA use, against our contemporary beliefs, a good SVG may not provide an inferior long-term survival. Further long-term contemporary studies with graft patency are required for validation of these conclusions. 

### Limitations

This is a single-centre retrospective study. We were not able to provide angiograms, graft patency data or analyse perioperative myocardial infarction. This is essentially a survival analysis with no follow-up data on cardiovascular events or repeat revascularisation. There was also no angiographic correlation to disease severity in the LAD and we did not have data on SYNTAX scores for all patients. Lastly, since the study included a 22-year study period, perioperative and postoperative management have changed and evolved over the time and could potentially affect the outcomes.

## 5. Conclusions

In this single-centre study, there was no difference in the propensity-matched cohorts for the use of SVG vs. LITA to the LAD. These results would need further substantiation and validation from other contemporary long-term studies.

## Figures and Tables

**Table 1:** Preoperative demographic and clinical characteristics for the overall cohort and the matched groups.

	Unmatched					Propensity Matched				
Variable	All *n* = 8327	LITA-LAD *n* = 6725	SVG-LAD *n* = 1602	*p* Value	SMD	All *n* = 2540	LITA-LAD N = 1270	SVG-LAD N = 1270	*p* Value	SMD
**Age, yrs**	69 (61.5–75.4)	68.2 (60.8–74.3)	73 (65.6–79.3)	<0.01	N/A	72.6 (66.1–78.1)	72.2 (66.9–77.4)	73 (65.9–79)	0.97	N/A
**Age, categories**				<0.01	−46.9				0.50	−0.2
**Age <=70 yrs**	4570 (54.9%)	3936 (58.5%)	634 (39.6%)			993 (39.1%)	488 (38.4%)	505 (39.8%)		
**Age 71–80 yrs**	3009 (36.1%)	2380 (35.3%)	629 (39.2%)			1124 (44.3%)	606 (47.7%)	518 (40.8%)		
**Age >80 yrs**	748 (9.0%)	409 (6.1%)	339 (21.1%)			423 (16.7%)	176 (13.9%)	247 (19.4%)		
**Male gender**	6716 (80.7%)	5491 (81.7)	1225 (76.5%)	<0.01	−12.7	1924 (75.7%)	957 (75.4%)	967 (76.1%)	0.64	1.9
**BMI, kg/m^2^**	27.7 (25.0–30.9)	27.7 (25.1–30.8)	27.6 (24.7–31.0)	0.66	−1.2	27.6 (24.9–31.0)	27.6 (24.9–31.1)	27.6 (24.7–31.0)	0.70	1.5
**Hypertension**	5567 (66.9%)	4452 (66.2%)	1115 (69.6%)	0.01	−8.5	1795 (70.7%)	898 (70.7%)	897 (70.6%)	0.97	0.2
**DM**	1591 (19.1%)	1267 (18.8%)	324 (20.2%)	0.16	−3.9	522 (20.6%)	269 (21.2%)	253 (19.9%)	0.43	3.2
**IDDM**	634 (7.6%)	501 (7.4%)	133 (8.3%)	0.25	N/A	191 (7.5%)	90 (7.1%)	101 (8.0%)	0.41	N/A
**Neuro dysfunction**	669 (8.0%)	153 (2.3%)	516 (32.2%)	0.01	−6.7	235 (9.3%)	113 (8.9%)	122 (9.6%)	0.54	−2.5
**Previous MI**	2996 (36.0%)	2452 (36.5%)	544 (34.0%)	0.07	5.1	883 (34.8%)	432 (34.0%)	451 (35.3%)	0.43	−3.1
**Smoking**	5696 (68.4%)	4607 (68.5%)	1089 (68.0%)	0.47	2.0	1740 (68.5%)	872 (68.7%)	868 (68.3%)	0.86	0.7
**COPD**	905 (10.9%)	666 (9.9%)	239 (14.9%)	<0.01	−15.7	394 (15.5%)	207 (16.3%)	187 (14.7%)	0.27	4.8
**CCS ≥3**	2659 (31.9%)	2211 (32.9%)	458 (28.6%)	<0.01	10.6	749 (29.5%)	390 (30.7%)	359 (28.3%)	0.17	5.3
**NYHA ≥3**	1697 (20.4%)	1402 (20.8%)	295 (18.4%)	0.04	5.8	466 (18.3%)	228 (18.0%)	238 (18.7%)	0.61	−2.0
**Previous PCI**	164 (2.0%)	127 (1.9%)	37 (2.3%)	0.27	−3.0	54 (2.1%)	26 (2.0%)	28 (2.2%)	0.78	−1.1
**CKD**	180 (2.2%)	123 (1.8%)	57 (3.6%)	<0.01	−10.7	71 (2.8%)	33 (2.6%)	38 (3.0%)	0.55	−2.4
**Extracardiac arteriopathy**	678 (8.1%)	529 (7.9%)	149 (9.3%)	0.04	−5.5	261 (10.3%)	132 (10.4%)	129 (10.2%)	0.85	0.8
**EuroSCORE II, mean**	4.0 (3.9–4.0)	3.7 (3.6–3.8)	5.1 (4.9–5.2)	<0.01	−48.7	5.0 (4.9–5.1)	5.0 (4.9–5.2)	5.0 (4.8–5.1)	0.67	1.7
**LMS disease**	2468 (29.6%)	1929 (28.7%)	539 (33.6%)	<0.01	−15.4	936 (36.9%)	462 (36.4%)	474 (37.3%)	0.62	−2.0
**Pre-op IABP**	451 (5.4%)	285 (4.2%)	166 (10.4%)	<0.01	−23.7	239 (9.4%)	126 (9.9%)	113 (8.9%)	0.38	4.0
**LVEF Categories**				<0.01	−12.8				0.87	0.6
**LVEF Good**	5905 (70.9%)	4856 (72.2%)	1049 (65.5%)			1687 (66.4%)	842 (66.3%)	845 (66.5%)		
**LVEF Mild**	1924 (23.1%)	1522 (22.6%)	402 (25.1%)			657 (25.9%)	344 (27.1%)	313 (24.6%)		
**LVEF Moderate/severe**	450 (5.4%)	322 (4.8%)	128 (8.0%)			174 (6.9%)	78 (6.1%)	96 (7.6%)		
**Unknown**	48 (0.6%)	25 (0.4%)	23 (1.4%)			22 (0.9%)	6 (0.5%)	16 (1.3%)		
**LVEF ≤30%**	450 (5.4%)	322 (4.8%)	128 (8.0%)	<0.01	N/A	174 (6.9%)	78 (6.1%)	96 (7.6%)	0.87	N/A
**Extent of vessel disease**				<0.01	−7.5				0.97	−1.4
**1 vessel >50% stenosis**	252 (3.0%)	225 (3.3%)	27 (1.7%)			57 (2.2%)	35 (2.8%)	22 (1.7%)		
**2 vessels >50% stenosis**	1415 (17.0%)	1150 (17.1%)	265 (16.5%)			464 (18.3%)	218 (17.2%)	246 (19.4%)		
**3 vessels >50% stenosis**	6030 (72.4%)	4904 (72.9%)	1126 (70.3%)			2019 (79.5%)	1017 (80.1%)	1002 (78.9%)		
**Unknown**	540 (6.6%)	446 (6.6%)	94 (5.9%)			16 (0.6%)	5 (0.4%)	11 (0.9%)		
**3-vessel CAD**	6030 (72.4%)	4904 (72.9%)	1126 (70.3%)			2019 (79.5%)	1017 (80.1%)	1002 (78.9%)	0.71	

Data presented as median (IQR) or *n* (percentage). BMI, body mass index; CAD, coronary artery disease; CCS, Canadian Cardiovascular Society; CKD, chronic kidney disease; COPD, chronic obstructive pulmonary disease; DM, diabetes mellitus; IABP, intra-aortic balloon pump; IDDM, insulin-dependent diabetes mellitus; LMS, left main stenosis; LVEF, left ventricular ejection fraction; MI, myocardial infarction; N/A, not applicable; NYHA, New York Heart Association; PCI, percutaneous intervention; SMD, standard mean difference; *p* < 0.05 is significant.

**Table 2:** Intraoperative characteristics in the unmatched and propensity matched cohorts.

	Unmatched				Propensity Matched			
Variable	All N = 8327	LITA-LAD *n* = 6725	SVG-LAD *n* = 1602	*p* Value	All *n* = 2540	LITA-LAD *n* = 1270	SVG-LAD *n* = 1270	*p* Value
CPB time	82 (65–99)	81 (65–99)	84 (70–101)	<0.01	83 (68–100)	82 (66–98)	84 (70–101)	0.03
XC time	47 (36–59)	47 (36–59)	46 (36–58)	0.39	46 (36–58)	47 (37–58)	46 (36–58)	0.70
Number of grafts				0.93				0.14
<3 grafts	1652 (19.8%)	1333 (19.8%)	319 (19.9%)		477 (18.8%)	224 (17.6%)	253 (19.9%)	
≥3 grafts	6675 (80.2%)	5392 (80.2%)	1283 (80.1%)		2063 (81.2%)	1046 (82.4%)	1017 (80.1%)	
Median number of grafts (LQ, UQ)	3 (3–3)	3 (3–4)	3 (3–3)	0.55	3 (3–3)	3 (3–4)	3 (3–3)	0.56

Data presented as median (IQR) or n (percentage). CPB, cardiopulmonary bypass time; LITA-LAD, left internal thoracic artery to left anterior descending artery; SVG-LAD; saphenous vein graft to left anterior descending artery; XC, cross-clamp time; *p* < 0.05 is significant.

**Table 3:** Postoperative characteristics in the unmatched and propensity-matched cohorts.

	Unmatched				Propensity Matched			
Variable	All N = 8327	LITA-LAD *n* = 6725	SVG-LAD *n* = 1602	*p* Value	All *n* = 2540	LITA-LAD *n* = 1270	SVG-LAD *n* = 1270	*p* Value
DSWI	23 (0.3%)	17 (0.3%)	6 (0.4%)	0.01	7 (0.3%)	3 (0.2%)	4 (0.3%)	0.71
New CVA	203 (2.4%)	157 (2.3%)	46 (2.9%)	0.21	72 (2.8%)	37 (2.9%)	35 (2.8%)	0.81
RRT	38 (0.5%)	25 (0.4%)	13 (0.8%)	0.02	17 (0.7%)	8 (0.6%)	9 (0.7%)	0.81
Return to theatre	128 (1.5%)	91 (1.4%)	37 (2.3%)	0.01	43 (1.7%)	14 (1.1%)	29 (2.3%)	0.02
LOS	9.02 (8.8–9.3)	8.8 (8.5–9.0)	10.1 (9.5–10.7)	<0.01	9.7 (9.3–10.2)	9.6 (8.9–10.4)	9.8 (9.3–10.4)	0.70
LOS >30 days	204 (2.4%)	152 (2.3%)	52 (3.2%)	0.02	77 (3.0%)	39 (3.1%)	38 (3.0%)	0.91
In-hospital mortality	80 (1.0%)	48 (0.7%)	32 (2.0%)	<0.01	30 (1.2%)	10 (0.8%)	20 (1.6%)	0.07
Composite endpoint	461 (5.5%)	349 (5.2%)	112 (7.0%)	0.01	163 (6.4%)	81 (6.4%)	82 (6.4%)	0.94
Median Survival, yrs (IQR)	16.1 (15.6–16.5)	16.5 (16.1–17.1)	12.9 (12.1–14.5)	<0.01	13.6 (12.9–14.4)	13.7 (12.9–14.7)	13.0 (12.1–14.9)	0.31

Data presented as median (IQR) or n (percentage). CVA, cerebrovascular accident; DSWI, deep sternal wound infection; LITA-LAD, left internal thoracic artery to left anterior descending artery; FU, follow-up; LOS, length of stay; RRT, renal replacement therapy; SVG-LAD; saphenous vein graft to left anterior descending artery; *p* < 0.05 is significant.

**Table 4:** Predictors of postoperative all-cause mortality with Cox regression model (matched Schoenfeld 0.19).

	Unmatched			Matched		
Variable	Hazard Ratio	CI	*p* Value	Hazard Ratio	CI	*p* Value
Age	1.01	1.01, 1.01	<0.001	1.08	1.07, 1.09	<0.001
Gender, Male	1.01	0.91, 1.12	0.908	0.80	0.68, 0.94	0.007
Angina	0.91	0.82, 1.00	0.041	0.97	0.83, 1.13	0.66
Dyspnoea	1.16	1.04, 1.28	0.006	1.14	0.97, 1.36	0.122
Previous MI	1.16	1.06, 1.26	0.001	1.10	0.95, 1.28	0.195
IDDM	1.69	1.53, 1.87	<0.001	1.70	1.44, 2.00	<0.001
Hypertension	1.23	1.12, 1.34	<0.001	1.25	1.07, 1.46	0.005
Smoking	1.35	1.23, 1.49	<0.001	1.33	1.14, 1.56	<0.001
COPD	1.34	1.18, 1.52	<0.001	1.28	1.07, 1.54	0.007
Extracardiac arteriopathy	1.26	1.11, 1.44	0.001	1.16	0.94, 1.43	0.163
Previous PCI	1.09	0.80, 1.47	0.592	1.39	0.93, 2.09	0.111
Preoperative renal dysfunction	2.26	1.80, 2.82	<0.001	1.68	1.18, 2.38	0.004
Preoperative neurological dysfunction	1.30	1.13, 1.48	0	1.09	0.88, 1.36	0.441
3 vessel disease	1.21	1.11, 1.32	<0.001	1.21	1.04, 1.42	0.016
LMS disease	0.99	0.91, 0.99	0.898	0.97	0.84, 1.13	0.724
BMI >30	0.98	0.97, 0.99	<0.001	0.99	0.98, 1.01	0.342
Surgery before 2010	0.93	0.88, 0.99	0.013	0.93	0.85, 1.01	0.091
≥3 grafts	0.90	0.84, 0.96	0.002	0.91	0.81, 1.03	0.134
CBP time	1.00	1.00, 1.01	0.053	1.00	1.00, 1.01	0.141
XC time	1.00	1.00, 1.00	0.019	0.99	0.98, 1.00	0.012
Log EuroSCORE	1.49	1.30, 1.72	<0.001	1.29	1.06, 1.57	0.011
RRT	2.16	1.18, 3.97	0.012	3.71	1.68, 8.16	0.001
DSWI	1.54	0.57, 4.11	0.393	2.53	0.81, 7.96	0.111
New CVA	1.49	1.18, 1.87	0.001	1.22	0.82, 1.81	0.334
SVG-to-LAD	0.90	0.80, 1.01	0.067	1.01	0.88, 1.16	0.905

BMI, body mass index; CBP, cardiopulmonary bypass time; CI, confidence interval; COPD, chronic obstructive pulmonary disease; CVA, cerebrovascular accident; DSWI, deep sternal wound infection; IDDM, insulin-dependent diabetes mellitus; LAD, left anterior descending artery; LMS, left main stenosis; MI, myocardial infarction; PCI; percutaneous intervention, RRT, renal replacement therapy; SVG; saphenous vein graft; XC, cross-clamp; *p* < 0.05 is significant.

## Data Availability

Data available on request. The data underlying this article will be shared on reasonable request to the corresponding author.

## Supplementary Materials

The following supporting information can be downloaded at: https://www.mdpi.com/article/10.3390/life14030385/s1, Table S1: STROBE Statement—checklist of items that should be included in reports of observational studies; Table S2: Regression analysis for in-hospital mortality (unmatched and propensity-matched), *p* < 0.2 entered into the model.
