# Supplementary material for: Left Internal Thoracic Artery versus Saphenous Vein Grafts to Left Anterior Descending Artery after Isolated Coronary Artery Bypass Surgery
Source: Life (Basel). 2024 Mar 14;14(3):385. doi: 10.3390/life14030385 (PMC10972088; doi:10.3390/life14030385)
Supplement: Supplementary file 1 [file life-14-00385-s001.zip › life-2910083-supplementary.pdf]

Supplementary Materials

Luthra S. et al. Left Internal Thoracic Artery versus Saphenous Vein Grafts to Left Anterior Descending Artery after Isolated Coronary Artery Bypass Surgery

Content:

Supplementary Table S1. STROBE Statement — checklist of items that should be included in reports of observational studies.

Supplementary Table S2. Regression analysis for in-hospital mortality (un-matched and propensity matched), p<0.2 entered into the model.

Supplementary Table S1. STROBE Statement — checklist of items that should be included in reports of observational studies.

	Item No.	Recommendation	Page (P) and Line (L)
**Title and abstract**	1	(*a*) Indicate the study's design with a commonly used term in the title or the abstract	P1, L1,2
		(*b*) Provide in the abstract an informative and balanced summary of what was done and what was found	P4, L79-103
**Introduction**			
Background/rationale	2	Explain the scientific background and rationale for the investigation being reported	P6, L133-142
Objectives	3	State specific objectives, including any prespecified hypotheses	P6, L144-153
**Methods**			
Study design	4	Present key elements of study design early in the paper	P6-7, L155-157
Setting	5	Describe the setting, locations, and relevant dates, including periods of recruitment, exposure, follow-up, and data collection	P7, L161-166
Participants	6	(*a*) *Cohort study*—Give the eligibility criteria, and the sources and methods of selection of participants. Describe methods of follow-up<br>*Case-control study*—Give the eligibility criteria, and the sources and methods of case ascertainment and control selection. Give the rationale for the choice of cases and controls.<br>*Cross-sectional study*—Give the eligibility criteria, and the sources and methods of selection of participants	P7, L167-170
		(*b*) *Cohort study*—For matched studies, give matching criteria and number of exposed and unexposed<br>*Case-control study*—For matched studies, give matching criteria and the number of controls per case	P7-8, L179-184
Variables	7	Clearly define all outcomes, exposures, predictors, potential confounders, and effect modifiers. Give diagnostic criteria, if applicable	P8, L193-201
Data sources/ measurement	8*	For each variable of interest, give sources of data and details of methods of assessment (measurement). Describe comparability of assessment methods if there is more than one group	P8, L193-201
Bias	9	Describe any efforts to address potential sources of bias	P8, 185-188
Study size	10	Explain how the study size was arrived at	N/A (retrospective study)

Continued on next page

Quantitative variables	11	Explain how quantitative variables were handled in the analyses. If applicable, describe which groupings were chosen and why	
Statistical methods	12	(*a*) Describe all statistical methods, including those used to control for confounding	P8, L181-184<br>P8, L185-188
		(*b*) Describe any methods used to examine subgroups and interactions	P8, L198-200
		(*c*) Explain how missing data were addressed	P7, L166
		(*d*) *Cohort study*—If applicable, explain how loss to follow-up was addressed<br>*Case-control study*—If applicable, explain how matching of cases and controls was addressed<br>*Cross-sectional study*—If applicable, describe analytical methods taking account of sampling strategy	N/A
		(*e*) Describe any sensitivity analyses	N/A
**Results**			
Participants	13*	(a) Report numbers of individuals at each stage of study—eg numbers potentially eligible, examined for eligibility, confirmed eligible, included in the study, completing follow-up, and analysed	P8, L205-206<br>P9, L213,214
		(b) Give reasons for non-participation at each stage	N/A
		(c) Consider use of a flow diagram	N/A
Descriptive data	14*	(a) Give characteristics of study participants (eg demographic, clinical, social) and information on exposures and potential confounders	Table 1
		(b) Indicate number of participants with missing data for each variable of interest	Table 1
		(c) *Cohort study*—Summarise follow-up time (eg, average and total amount)	P8, L206,207<br>Table 1
Outcome data	15*	*Cohort study*—Report numbers of outcome events or summary measures over time	P9, L215-225 (perioperative)<br>P9-10, L226-239 (survival)
		Case-control study—Report numbers in each exposure category, or summary measures of exposure	
		Cross-sectional study—Report numbers of outcome events or summary measures	

Main results	16	(*a*) Give unadjusted estimates and, if applicable, confounder-adjusted estimates and their precision (eg, 95% confidence interval). Make clear which confounders were adjusted for and why they were included	Table 2 (perioperative outcomes and survival - unmatched and propensity matched outcomes)
		(*b*) Report category boundaries when continuous variables were categorized	Table 1, 3
		(*c*) If relevant, consider translating estimates of relative risk into absolute risk for a meaningful time period	Table 3<br>Supplementary table 2
Other analyses	17	Report other analyses done—eg analyses of subgroups and interactions, and sensitivity analyses	P10, L240-244
**Discussion**			
Key results	18	Summarise key results with reference to study objectives	P13, L317-319
Limitations	19	Discuss limitations of the study, taking into account sources of potential bias or imprecision. Discuss both direction and magnitude of any potential bias	P13, L312.315
Interpretation	20	Give a cautious overall interpretation of results considering objectives, limitations, multiplicity of analyses, results from similar studies, and other relevant evidence	P12, L307-311
Generalisability	21	Discuss the generalisability (external validity) of the study results	P11-12, L279-306
**Other information**			
Funding	22	Give the source of funding and the role of the funders for the present study and, if applicable, for the original study on which the present article is based	P2. L27

*Give information separately for cases and controls in case-control studies and, if applicable, for exposed and unexposed groups in cohort and cross-sectional studies.

Note: An Explanation and Elaboration article discusses each checklist item and gives methodological background and published examples of transparent reporting. The STROBE checklist is best used in conjunction with this article (freely available on the Web sites of PLoS Medicine at http://www.plosmedicine.org/, Annals of Internal Medicine at http://www.annals.org/, and Epidemiology at http://www.epidem.com/). Information on the STROBE Initiative is available at www.strobe-statement.org.

Supplementary Table S2. Regression analysis for in-hospital mortality (un-matched and propensity matched), *p*<0.2 entered into the model.

	**Unmatched**						**Propensity matched**					
	Univariable			Multivariable			Univariable			Multivariable		
**Variable**	OR	CI	P value	OR	CI	P value	OR	CI	P value	OR	CI	P value
Vein graft	2.84	1.81-4.45	<0.001	1.71	1.00-2.94	0.05	2.02	0.94-4.32	0.07	2.03	0.91-4.54	0.08
Age	1.10	1.07-1.14	<0.001	1.07	1.03-1.11	<0.001	1.09	1.04-1.15	0.001	1.07	1.00-1.14	0.03
Gender	1.21	0.72-2.05	0.48	0.90	0.48-1.66	0.73	0.95	0.41-2.22	0.91	0.75	0.30-1.84	0.53
Angina, CCS 3-4	2.81	1.80-4.40	<0.001	2.12	1.24-3.63	0.006	4.21	1.99-8.89	<0.001	2.74	1.15-6.51	0.02
Dyspnoea, NYHA 3-4	2.21	1.38-3.52	0.001	1.34	0.78-2.32	0.29	3.98	1.93-8.22	<0.001	2.23	0.99-5.04	0.05
Previous MI	2.70	1.71-4.27	<0.001	1.66	0.98-2.80	0.06	3.82	1.78-8.19	0.001	1.77	0.75-4.17	0.19
Hypertension	0.74	0.47-1.17	0.20	0.67	0.41-1.12	0.13	1.14	0.51-2.58	0..75			
Smoking history	1.48	0.87-2.51	0.15	1.35	0.75-2.41	0.31	1.27	0.56-2.86	0.57			
Extracardiac arteriopathy	2.37	1.29-4.33	0.005	1.12	0.55-2.27	0.76	2.21	0.90-5.46	0.09	1.10	0.40-3.04	0.85
Log EuroSCORE >10%	7.55	4.80-11.86	<0.001	2.00	0.96-4.18	0.07	8.51	4.07-17.82	<0.001	3.07	1.05-8.97	0.04
BMI ≥30 kg/m²	0.52	0.30-0.91	0.02	0.61	0.33-1.16	0.13	0.44	0.17-1.14	0.09	0.57	0.21-1.57	0.28
LVEF ≤30%	6.90	4.18-11.38	<0.001	2.90	1.53-4.40	0.001	5.86	2.64-12.98	<0.001	2.03	0.77-5.33	0.15
CKD	7.62	3.96-14.66	<0.001	3.71	1.72-8.02	0.001	7.41	2.75-19.95	<0.001	3.09	0.96-9.99	0.06
Surgery after 2010	0.51	0.32-0.82	0.006	0.52	0.30-0.91	0.02	0.41	0.20-0.87	0.02	0.57	0.25-1.29	0.18
≥3 grafts	0.99	0.57-1.72	0.97				0.63	0.28-1.43	0.27			
Neurological disease	1.28	0.61-2.66	0.52				1.09	0.33-3.62	0.89			
IDDM	2.16	1.16-4.02	0.02	2.37	1.18-4.79	0.02	2.50	0.95-6.60	0.07	1.99	0.64-6.24	0.24
3-vessel CAD	1.32	0.78-2.23	0.31				1.29	0.49-3.40	0.60			
LMS	1.21	0.74-1.98	0.46				1.31	0.64-2.72	0.46			

Critical preoperative state	2.31	1.39-3.85	0.001	0.92	0.48-1.78	0.81	2.37	1.10-5.10	0.03	0.97	0.38-2.50	0.96

BMI, body mass index; CAD, coronary artery disease; CCS, Canadian Cardiovascular Society; CKD, chronic kidney disease; IDDM, insulin-dependent diabetes mellitus; LMS, left main stenosis; LVEF, left ventricular ejection fraction; MI, myocardial infarction; NYHA, New York Heart Association; $p<0.05$ is significant.
